# The impact of mixed exposure to PM_2.5_ components before and during pregnancy on renal function in pregnant women

**DOI:** 10.3389/fpubh.2026.1770917

**Published:** 2026-05-20

**Authors:** Wu Yan, Xuemei Guo, Yiming Du, Beibei Gao, Rufeng Lin, Xu Wang

**Affiliations:** 1 Department of Children Health Care, Children's Hospital of Nanjing Medical University, Nanjing, China; 2 Department of Hematology and Oncology, Children's Hospital of Nanjing Medical University, Nanjing, China; 3 Pediatric Department, Zhongda Hospital, Southeast University, Nanjing, China; 4 State Key Laboratory of Reproductive Medicine and Offspring Health, Center for Global Health, School of Public Health, Nanjing Medical University, Nanjing, China; 5 Key Laboratory of Modern Toxicology of Ministry of Education, School of Public Health, Nanjing Medical University, Nanjing, China; 6 Clinical Medical Research Center, Children's Hospital of Nanjing Medical University, Nanjing, China

**Keywords:** creatinine, mixed exposure, PM2.5 components, renal function, urea nitrogen

## Abstract

**Background:**

Particulate matter 2.5 (PM_2.5_) has been associated with numerous health effects, yet there is limited understanding of specific effects on kidney health in women prior and during pregnancy.

**Methods:**

A cohort study was conducted. The satellite spatiotemporal inversion model combined with aerosol optical depth estimated the PM_2.5_ exposure levels and six main components before (4 weeks, 8 weeks, and 12 weeks), and during (13 weeks) pregnancy. Blood urea nitrogen (BUN), serum creatinine (SCr), and uric acid (UA) were assessed at 13 weeks of gestation. Multiple linear regression and weighted quantile sum regression models were used to investigate the nephrotoxic effects of PM_2.5_ and its components.

**Results:**

The associations between PM_2.5_ and BUN, SCr, and UA at 13 weeks of gestation were explored in 1,499, 1,508, and 699 participants, respectively. Maternal SCr increased by 2.44 μmol/L (95%CI:1.31, 3.58) at 13 weeks of gestation and 4.56 μmol/L (95%CI: 3.68, 5.44) at 4 weeks before pregnancy, respectively. The primary contributors to SCr from PM_2.5_ components were organic matter and soil dust at 4 weeks before pregnancy, and for soil dust and black carbon at 13 weeks of gestation.

**Conclusion:**

Mixed exposure to PM_2.5_ components was in connection with renal function in pregnant women, with potential health implications for women as well as infants.

## 1 Introduction

Particulate matter 2.5 (PM_2.5_) is a mixture of solid particles and liquid droplets that can be formed directly from burning of fossil fuel, industrial processing, construction activities, and various transportation sources, or indirectly through numerous chemical and physical processes in the atmosphere. The composition of PM_2.5_ varies greatly in different regions due to different natural sources and human production activities, mainly including organic matter (OC), black carbon (BC), sulfate (${\text{SO}}_{4}^{2-}$), ammonium salt (${\text{NH}}_{4}^{+}$), nitrate (${\text{NO}}_{3}^{-}$), and soil dust (SOIL) (1). PM_2.5_ can enter gas-exchange areas in the lungs, and further enter the circulatory system through the respiratory barrier, thus spreading to the whole body. Therefore, PM_2.5_ can cause a wide range of adverse health effects on multiple systems, for example, the cardiovascular system, respiratory system, nervous system, and immune system (2–5). Recently, the influence of PM_2.5_ exposure on the renal system has gradually attracted attention (6–8).

Previous studies suggested that PM_2.5_ exposure could affect the renal function in the general population (9). Due to the special physiological metabolic state during pregnancy, pregnant women are often show more sensitive to environmental exposure, and physiological homeostasis becomes more vulnerable. A large population-based study in China reported that PM_2.5_ exposure during pregnancy was associated with altered renal function indicators among pregnant women (10).

However, despite the valuable insights provided by previous studies, several gaps remain in the current understanding. While most available evidence has primarily focused on exposure during pregnancy, whereas the pre-pregnancy period—which included the gametogenic process, and early embryonic development is also considered a sensitive window for environmental harmful factors (11). Moreover, although PM_2.5_ is composed of a mixture of multiple chemical components, many studies have mainly examined PM_2.5_ mass concentration without considering component-specific effects (12). Additionally, while the renal system is recognized as a key target organ for environmental toxicants (13, 14), and impaired renal function during pregnancy has a short-term and long-term adverse impact on maternal and offspring health (15), the combined effects of PM_2.5_ components as a mixture remain insufficiently characterized in pregnant populations.

Therefore, in this cohort study, we utilized combined single and mixed exposure models to examine the connections between PM_2.5_ exposure prior to and during pregnancy and renal function at 13 weeks of gestation. We hypothesized that exposure to PM_2.5_ and its specific components before and during pregnancy is correlated with impaired renal function.

## 2 Methods

### 2.1 Research design and participants

A total of 1, 715 participants were recruited from two hospitals in Jiangsu Province, China from 2014 to 2015. The inclusion criteria specified pregnant women over 18 years of age, residing in the city where the hospital is situated or nearby suburbs and towns, and not actively smoking before and during pregnancy. Exclusion criteria included women with pre-existing kidney disease, as well as women with other risk factors for kidney disease such as diabetes and cardiovascular disease. Questionnaires were conducted at 13 weeks of gestation to collect demographic information including age, BMI, education, passive smoking, alcohol consumption, household income, twin, parity, and assisted reproduction. Of 1,715 eligible women who attended prenatal clinic visits, 1,499 had complete information on blood urea nitrogen (BUN), 1,508 on serum creatinine (SCr), and 699 on uric acid (UA) measured at 13 weeks of gestation.

### 2.2 Evaluation of maternal exposure to PM_2.5_ and its components

We estimated PM_2.5_ and its six components, namely OC, BC, ${\text{SO}}_{4}^{2-}$, ${\text{NH}}_{4}^{+}$, ${\text{NO}}_{3}^{-}$, and SOIL before pregnancy (at 4 weeks, 8 weeks, and 12 weeks) and during pregnancy at 13 weeks of gestation based on each participant's residential address. We used the monthly average value from the exposure assessment model to represent the daily exposure within each month; that is, for each specific day in a month, the same monthly average value was assigned. To estimate exposures during specific preconception windows (e.g., 4, 8, and 12 weeks before pregnancy), we first determined these windows based on the last menstrual period of each participant. If a specific exposure window (such as the 4 weeks before conception) spanned 2 calendar months, the exposure for that window was calculated as the weighted average of the monthly exposure values, with weights corresponding to the number of days falling within each month. For example, if a 4-week window includes 10 days in April and 18 days in May, the exposure for this window equals [(April monthly average × 10 days) + (May monthly average × 18 days)] ÷ 28 days. This approach allowed us to approximate daily exposure during the relevant windows using the available monthly estimates.

The average PM_2.5_ and its six components were evaluated employing the model V4.CH.02 exploited by Dalhousie University described in detail elsewhere (1). Concisely, PM_2.5_ measurements were obtained by the combination of Aerosol Optical Depth (AOD) data retrievals from Moderate Resolution Imaging Spectroradiometer (MODIS), Multi-angle Imaging Spectroradiometer (MISR), Sea-Viewing Wide Field-of-View Sensor (SeaWiFS) instruments, and atmospheric chemistry and composition transmission model (Goddard Earth Observing System-Chemistry, GEOS-Chem).

Geographically Weighted Regression (GWR) was used to calibrate regional ground observations of the total mass and component of PM_2.5_ at a spatial resolution of 0.1° × 0.1°. Using an expanded PM_2.5_ measurement network, a comprehensive geophysical statistical estimate of PM_2.5_ over China was performed. The model was cross-verified out of the sample to check the consistency between the simulated data and the ground time monitoring data and the R^2^ = 0.78 (16). GEOS-Chem was applied to partition PM_2.5_ into its components; OC, BC, ${\text{SO}}_{4}^{2-}$, ${\text{NH}}_{4}^{+}$, ${\text{NO}}_{3}^{-}$, and SOIL.

### 2.3 Assessment of maternal renal function

Fasting blood samples were collected in the morning from pregnant women at 13 weeks of gestation during routine check-ups at an antenatal clinic. Blood samples were stored frozen at 2°C until analyzed. The blood urea nitrogen (BUN), serum creatinine (SCr), and uric acid (UA) levels of pregnant women were measured by Beckman AU5800 automatic biochemical analyzer at 13 weeks of gestation as the indicators of kidney function. The reference intervals are BUN 2.60–7.50 mmol/L; SCr 33.90–90.20 μmol/L; UA 90.00–430.00 μmol/L.

### 2.4 Covariates

Covariates were selected based on the prior knowledge from the scientific literature (10, 17) and visualized in the directed acyclic graph (DAG) using the DAGitty website as shown in Supplementary Figure S1. The minimum adjustment sets of covariates selected were maternal age (<30, 30–35, >35), pre-pregnancy body mass index (BMI; <18.5 kg/m^2^, 18.5–24.9 kg/m^2^, ≥25 kg/m^2^), maternal education (<high school, high school, >high school), passive smoking (no, <0.5 h/day, 0.5–1 h/day, >1 h/day), alcohol consumption (no, yes), household income (<30 thousand yuan, 30–50 thousand yuan, 50–100 thousand yuan, 100–200 thousand yuan, >200 thousand yuan), twin (no, yes), parity (0, 1, 2), and assisted reproduction (no, yes).

### 2.5 Statistical analysis

The PM_2.5_ exposure level of the included population was presented as mean ± SD, and the comparison of PM_2.5_ with different demography characteristics was performed by *t-*test or one-way ANOVA. PM_2.5_, components, and renal function related indicators in different periods were expressed in the form of quantiles. Multiple linear regression and weighted quantile sum (WQS) regression models were used to examine the association between PM_2.5_ and its components and renal function at 13 weeks of gestation, and the characteristics of different statistical methods were integrated to interpret the results.

Firstly, multiple linear regression models were utilized to evaluate the connection between PM_2.5_ and BUN, SCr, and UA. Model 1 was unadjusted to examine the association between the PM_2.5_ components and renal function without accounting for potential confounders. Model 2 was adjusted for age, BMI, education, passive smoking, alcohol consumption, household income, twin, parity, and assisted reproduction. We used prior knowledge from the scientific literature to decide whether to select covariates for statistical analysis. WQS regression model was utilized to comprehensively explore the relationship between PM_2.5_ components with related renal function indicators. WQS regression model constructed a single score (WQS) through a weighted additive index of PM_2.5_ and its six components into quantiles, in such a way that PM_2.5_ and its 6 components with weak effect on the renal function assigned a lower weight in the WQS index. The score is incorporated in a regression to assess the joint effect of the mixture of PM_2.5_ components on renal function among pregnant women. The detailed methods are presented elsewhere (18–20).

Data was randomly allocated into two datasets to improve the reliability of the results, with 40% training set and 60% validation set. The weights of each component were calculated in the training set, the WQS score was calculated in the validation set, and the relationship between the WQS score and renal function was examined. A *P*-value < 0.05 based on two-tailed test results was considered statistical significance. All analyses were conducted with R Studio (version 4.1.1). WQS regression model was analyzed using the R packages “gWQS” (version 3.0.4).

To evaluate the robustness of the WQS model findings, we performed sensitivity analyses using quantile g-computation (Qgcomp). Qgcomp is a complementary mixture modeling approach that estimates both positive and negative weights for individual components, thereby reflecting potential antagonistic or synergistic interactions among PM_2.5_ components. The Qgcomp models were adjusted for the same covariates as those used in the WQS analysis. Consistency between the WQS and Qgcomp results would strengthen the robustness of the observed associations, while discrepancies may provide insights into the complex interactions among PM_2.5_ components. The Qgcomp analysis was conducted using the R package “qgcomp” (version 2.18.7).

## 3 Results

### 3.1 Characteristics of participants before and during pregnancy

Table 1 shows the characteristics of participants and their exposure to PM_2.5_ 4 weeks before pregnancy. Among all subjects, the maternal age under thirty accounted for the largest proportion (63.8%), and no significant difference can be seen in exposure between different age groups. 1,092 (72.4%) women had a BMI between 18.5 and 24.9 kg/m^2^ in the 4th week before pregnancy, and there was no significant difference in exposure among different BMI groups. The majority of the participants 963 (63.9%) reported education above high school, and the distribution of PM_2.5_ was significantly different among the education level groups (*P* = 0.002). There were also significant differences in PM_2.5_ exposure concentrations among twins (*P* = 0.003), parity (*P* = 0.013), and assisted reproduction (*P* = 0.044). Supplementary Table S1 shows the characteristics of participants and their exposure to PM_2.5_ at 13 weeks of gestation. During pregnancy, the distribution of PM_2.5_ was significantly different in maternal age (*P* = 0.028) and marginal in maternal education (*P* = 0.05). Supplementary Table S2 presents the baseline characteristics of pregnant women included in and excluded from the UA analysis.

**Table 1:** The characteristics of participants, and their exposure to PM_2.5_ 4 weeks before pregnancy (*N* = 1,508).

Characteristic	Participants	PM_2.5_ (μg/m^3^)	*F*	*P*-values
		*n* (%)	(MEAN ±SD)	
Maternal age (years)				
<30	962 (63.8)	60.265 ± 0.39	0.422	0.656
30–35	472 (31.3)	60.475 ± 0.49		
>35	74 (4.9)	60.715 ± 0.87		
Pre-pregnancy BMI (kg/m^2^)				
<18.5	260 (17.2)	60.725 ± 0.49	0.763	0.466
18.5–24.9	1,092 (72.4)	60.265 ± 0.46		
≥25	156 (10.3)	60.35 ± 0.26		
Maternal education				
<high school	176 (11.7)	59.475 ± 0.07	6.190	**0.002**
high school	369 (24.5)	59.825 ± 0.11		
>high school	963 (63.9)	60.715 ± 0.60		
Alcohol consumption				
No	1,455 (96.5)	60.325 ± 0.44	−0.929	0.353
Yes	53 (3.5)	61.035 ± 0.57		
Household income (yuan)				
<30 thousand	22 (1.5)	60.194 ± 0.97	1.263	0.282
30–50 thousand	106 (7)	59.714 ± 0.77		
50–100 thousand	355 (23.5)	60.055 ± 0.24		
100–200 thousand	823 (54.6)	60.625 ± 0.61		
> 200 thousand	202 (13.4)	60.095 ± 0.45		
Passive smoking				
No	1,133 (75.1)	60.265 ± 0.42	1.391	0.244
<0.5 h/day	296 (19.6)	60.725 ± 0.49		
0.5–1 h/day	63 (4.2)	59.745 ± 0.43		
>1 h/day	16 (1.1)	62.126 ± 0.26		
Twin				
No	1,481 (98.2)	60.295 ± 0.43	−3.022	**0.003**
Yes	27 (1.8)	63.475 ± 0.00		
Parity (times)				
0	1,213 (80.4)	60.555 ± 0.57	4.364	**0.013**
1	288 (19.1)	59.534 ± 0.80		
2	7 (0.5)	58.864 ± 0.61		
Assisted reproduction				
No	1,478 (98)	60.315 ± 0.42	−2.018	**0.044**
Yes	30 (2)	62.336 ± 0.20		

No 2. Less than 0.5 h/day 3. 0.5–1 h/day 4. More than 1 h/day. Bold values indicate statistical significance (*P* < 0.05).

### 3.2 Distribution of PM_2.5_ and renal function indicators

Supplementary Table S3 displays the percentiles and ranges of exposure to PM_2.5_ and its components (OC, BC, ${\text{SO}}_{4}^{2-}$, ${\text{NH}}_{4}^{+}$, ${\text{NO}}_{3}^{-}$, and SOIL) at 4, 8, and 12 weeks before pregnancy, and at 13 weeks of gestation. The range of PM_2.5_ was 34.00–89.25μg/m^3^, with a median concentration of 61.00μg/m^3^ at 13 weeks of gestation. PM_2.5_ levels were recorded at 46.89–75.50 μg/m^3^, 25.00–111.00 μg/m^3^, and 28.00–102.00 μg/m^3^ at 4, 8, and 12 weeks before pregnancy, with median concentrations of 59.00 μg/m^3^, 54.50 μg/m^3^ and 54.50 μg/m^3^, respectively. Supplementary Table S4 presents the percentile and range of BUN, SCr, and UA concentrations at 13 weeks of gestation. The range of BUN was 1.00–9.00 mmol/L, with a median concentration of 2.82 mmol/L. For SCr, the range was 3.55–208.50 μmol/L, and the median concentration was 43.90 μmol/L. UA exhibited a range of 57.70–478.30 μmol/L, with a median concentration of 195.4 μmol/L.

### 3.3 Multiple linear regression to examine the association between PM_2.5_ and renal function indicators

Table 2 shows the results of associations between PM_2.5_ and the indicators of renal function including BUN, SCr, and UA. Maternal PM_2.5_ exposure at 13 weeks of gestation was positively associated with SCr during the same period (β = 0.07; 95%CI: 0.02, 0.12). The exposure to PM_2.5_ at 4 weeks before pregnancy is positively associated with both SCr and UA at 13 weeks of gestation, and its exposure concentration‘s each 1 μg/m^3^ increase was connected with 0.39 μmol/L increase in SCr (95%CI: 0.26, 0.52), and each its 1 μg/m^3^ increase was connected with a 1.12 μmol/L increase in UA (95%CI: 0.29, 1.94). PM_2.5_ exposure at 8 (β = 0.003; 95%CI: 0.001, 0.005) and 12 weeks (β = 0.003; 95%CI: 0, 0.005) before pregnancy was positively correlated with BUN, and exposure at 12 weeks before pregnancy also significantly increased UA (β = 0.31; 95%CI: 0.051, 0.56).

**Table 2:** The associations between PM_2.5_ levels and renal function among pregnant women.

Renal function indicators	Model 1^a^			Model 2^b^		
	β	*95% CI*	*P-*values	β	*95% CI*	*P-*values
At 13 weeks of gestation						
BUN	0.001	(−0.002, 0.004)	0.468	0.001	(−0.002, 0.004)	0.473
SCr	0.080	(0.029, 0.131)	**0.002**	0.070	(0.020, 0.121)	**0.006**
UA	−0.196	(−0.450, 0.058)	0.131	−0.184	(−0.439, 0.072)	0.158
4 weeks before pregnancy						
BUN	−0.001	(−0.009, 0.006)	0.761	−0.002	(−0.009, 0.006)	0.674
SCr	0.438	(0.312, 0.565)	**<0.001**	0.391	(0.265, 0.516)	**<0.001**
UA	1.235	(0.431, 2.039)	**0.003**	1.117	(0.295, 1.938)	**0.008**
8 weeks before pregnancy						
BUN	0.003	(0.001, 0.005)	**0.014**	0.003	(0.001, 0.005)	**0.015**
SCr	0.041	(0.002, 0.08)	**0.039**	0.036	(−0.002, 0.074)	0.063
UA	0.222	(−0.009, 0.452)	0.059	0.184	(−0.049, 0.417)	0.121
12 weeks before pregnancy						
BUN	0.003	(0, 0.005)	**0.030**	0.003	(0, 0.005)	**0.035**
SCr	0.037	(−0.005, 0.079)	0.088	0.031	(−0.011, 0.073)	0.144
UA	0.341	(0.089, 0.594)	**0.008**	0.306	(0.051, 0.562)	**0.019**

BUN, blood urea nitrogen (mmol/L); SCr, serum creatinine (μmol/L); UA, uric acid (μmol/L); Cl, confidence interval. ^a^unadjusted model; ^b^ adjusted model, adjusted for age, BMI, education, passive smoking, alcohol consumption, household income, twin, parity, and assisted reproduction. In BUN, SCr, and UA model analyses, there were 1,499, 1,508, and 699 participants, respectively. Renal function indicators were measured at 13 weeks of gestation. Bold values indicate statistical significance (*P* < 0.05).

### 3.4 WQS regression to examine the association between PM_2.5_ components and renal function indicators

The associations between the mixture of PM_2.5_ and renal function indicators were analyzed by WQS. The adjusted results demonstrated a significant positive connection between the WQS index of PM_2.5_ component exposure and SCr at 13 weeks of gestation, which means that SCr increased by 2.44 μmol/L (95% CI:1.31, 3.58) when the WQS index increases each unit. Similar results also appeared at 4 weeks before the pregnancy (β = 4.56, 95% CI: 3.68, 5.44; Table 3).

**Table 3:** Associations between the mixture of PM_2.5_ components and renal function among pregnant women using WQS.

Renal function indicators	Model 1^a^			Model 2^b^		
	β	*95% CI*	*P-*values	β	*95% CI*	*P-*values
At 13 weeks of gestation						
BUN	0.039	(−0.035, 0.112)	0.301	0.031	(−0.037, 0.1)	0.372
SCr	2.543	(1.401, 3.684)	**<0.001**	2.444	(1.308, 3.579)	**<0.001**
UA	2.270	(−3.996, 8.536)	0.478	1.379	(−5.048, 7.806)	0.674
4 weeks before pregnancy						
BUN	−0.011	(−0.082, 0.06)	0.766	−0.003	(−0.058, 0.052)	0.926
SCr	4.849	(3.977, 5.721)	**<0.001**	4.562	(3.685, 5.439)	**<0.001**
UA	3.870	(−1.097, 8.837)	0.127	3.373	(−1.712, 8.458)	0.194
8 weeks before pregnancy						
BUN	0.029	(−0.025, 0.082)	0.292	0.033	(−0.021, 0.088)	0.228
SCr	0.364	(−0.784, 1.512)	0.535	−0.108	(−1.13, 0.914)	0.836
UA	5.794	(−0.188, 11.776)	0.058	5.029	(−0.845, 10.902)	0.094
12 weeks before pregnancy						
BUN	0.020	(−0.03, 0.069)	0.430	0.021	(−0.029, 0.071)	0.406
SCr	0.139	(−1.012, 1.29)	0.813	−0.343	(−1.215, 0.53)	0.441
UA	5.651	(0.759, 10.543)	**0.024**	4.887	(−0.038, 9.813)	0.053

BUN, blood urea nitrogen (mmol/L); SCr, serum creatinine (μmol/L); UA, uric acid (μmol/L); Cl, confidence interval. ^a^unadjusted model. ^b^adjusted model, adjusted for age, BMI, education, passive smoking, alcohol consumption, household income, twin, parity, and assisted reproduction. In BUN, SCr, and UA model analyses, there were 1,499, 1,508, and 699 participants, respectively. Bold values indicate statistical significance (*P* < 0.05).

**Figure 1:**
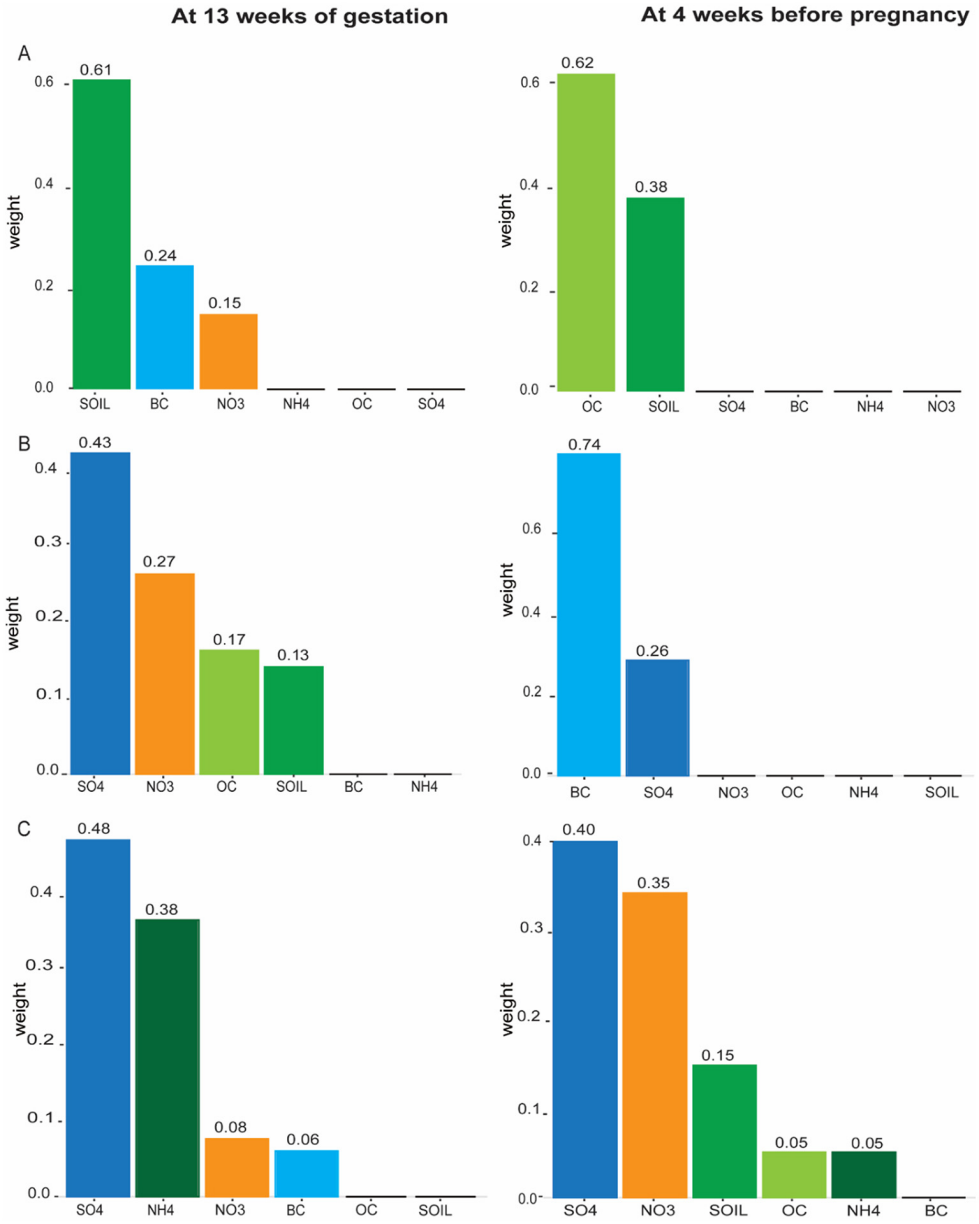
WQS regression model weights of the association between PM_2.5_ component and renal function indicators at 13 weeks of gestation and 4 weeks before pregnancy. **(A)** Serum creatinine (SCr). **(B)** Blood urea nitrogen (BUN). **(C)** Uric acid (UA).

Figure 1 shows the weight of each component of PM_2.5_ in the WQS model at 13 weeks of gestation and 4 weeks before pregnancy. For SCr, at 13 weeks of gestation exposure model, the chemical with the highest weight was SOIL (weighted 0.61), followed by BC (weighted 0.24) and ${\text{NO}}_{3}^{-}$ (weighted 0.15). The sum of the weights of OC (weighted 0.62) and SOIL (weighted 0.38) in the 4 weeks before pregnancy model was approximately 1, so the remaining small weights component were scarcely represented (Figure 1A). The weight of each PM_2.5_ component at 13 weeks of gestation and 4 weeks before pregnancy for BUN and UA is shown in Figures 1B, C, respectively.

### 3.5 Sensitivity analysis

We employed Qgcomp as a sensitivity analysis to assess the associations between exposure to PM_2.5_ component mixtures and renal function in pregnant women. The results are presented in Supplementary Table S5. Consistent with the WQS model, the Qgcomp analysis also revealed a significant positive association between PM_2.5_ mixture exposure and SCr at 4 weeks before pregnancy (ψ = 3.392, 95% CI: 2.281, 4.503, *P* < 0.001), closely aligning with the WQS estimates (β = 4.562, *P* < 0.001). This consistency reinforces the robustness of the observed effect during this exposure window. For other time points and renal function indicators, the Qgcomp results showed generally similar trends compared to the WQS model, with consistent effect directions for most outcomes. Minor discrepancies in statistical significance were observed for a few indicators, which may be attributable to inherent methodological differences between the two modeling approaches, particularly in weight estimation and underlying assumptions. Overall, the sensitivity analysis supports the primary findings derived from the WQS model and further confirms the robustness of our conclusions.

## 4 Discussion

This study used the satellite spatiotemporal inversion model combined with AOD to estimate maternal exposure to PM_2.5_ and its major components during several preconception windows (4, 8, and 12 weeks before pregnancy; 4 weeks, 8 weeks, and 12 weeks) and in early pregnancy (13 weeks of gestation). Multiple linear regression models were applied to examine the associations between PM_2.5_ mass concentrations and renal function indicators at 13 weeks of gestation, and WQS regression model was used to evaluate joint associations of PM_2.5_ components mixtures and to identify the main contributing components. Overall, we observed that higher exposure to PM_2.5_ and its components mixtures during both the preconception period and early pregnancy was associated with changes in maternal renal function indicators. Notably, the associations for preconception exposure windows provide clearer temporality compared with the concurrent exposure analysis at 13 weeks. Among the PM_2.5_ components, OC, BC, and SOIL consistently showed the greatest contributions in the mixture models.

The median exposure level of PM_2.5_ before (4 weeks, 8 weeks, and 12 weeks) pregnancy and at 13 weeks of gestation was 54.50–61.00μg/m^3^. The PM_2.5_ exposure levels of the participants in this research were more than 10 times higher than the World Health Organization's (WHO) 2021 average annual recommended PM_2.5_ concentration of 5.00 μg/m^3^, as well as the global urban average of 22.00 μg/m^3^ (21). The previous study monitored and sampled the PM_2.5_ level in the main urban area of Jiangsu Province for 7 years and analyzed its chemical composition changes, and the major components of PM_2.5_ are consistent with those calculated by our model. In addition, most components showed a decreasing trend with the decrease in PM_2.5_ density.

Multiple linear regression and WQS regression models were used to find whether PM_2.5_ exposure or multi-component exposure 4 weeks before and during pregnancy was positively correlated with the increased SCr at 13 weeks of gestation. The WQS regression model helps to analyze the health effects of mixed chemical exposure and establish the health burden of combined chemical exposure based on bootstrap sampling (22–24). In our analysis, the WQS index showed that joint exposure to the six PM_2.5_ components before and during pregnancy was significantly positively associated with maternal SCr, which is consistent with our results in Multiple linear regression models. In agreement with our findings, a previous study conducted in China showed that PM_2.5_ exposure during pregnancy was associated with decreased renal function (10).

It has been suggested that SCr should be used to assess renal function during pregnancy because estimated glomerular filtration rate (eGFR) is invalid for use during pregnancy (25). This can be explained that during pregnancy women experience increased plasma flow and filtration fraction changing dynamics (26), leading to an increase in eGFR up to 50% and decreased SCr concentrations. In addition, research conducted among pregnant women in Canada noted a decline in a mean concentration of SCr from 60 μmol before pregnancy to a nadir of 47 μmol/L between 16 and 32 weeks of gestation, which then rose to 64 μmol/L in the 1st weeks of post-partum before returning to its original concentration before pregnancy by 18 weeks postpartum (27).

A previous study has shown that increased PM_2.5_ concentration was related to the risk of developing chronic kidney disease (CKD) among non-pregnant adults (28). Another study also linked short exposure to PM_2.5_ with adverse effect on renal health (29). However, other studies found no significant correlation between PM_2.5_ and CKD (30). A national population-based longitudinal cohort study of participants aged 65 years or older in the United States found a connection between PM_2.5_ exposure and the risk of hospitalization for acute kidney injury (AKI), even at low concentrations of air pollution (31). The age of participants and differences in renal function evaluation indicators may lead to inconsistent conclusions. On the other hand, the above studies cannot be extended to pregnant individuals. Our study provides important evidence that exposure to PM_2.5_ and its key components is related to renal function indicators in pregnant women, which can fill the gap in evidence of the effects of air pollution on renal function in special populations.

The mechanism of renal function changes induced by PM_2.5_ exposure has not been fully understood. However, it is plausible that exposure to PM_2.5_ may aggravate renal oxidative stress, increase renal lipid peroxidation levels, inflammation, and DNA damage, resulting in renal cortical damage and interstitial fibrosis (32). Some studies also suggest that PM_2.5_ exposure may cause chronic renal insufficiency by altering hemodynamic pathways (33). PM_2.5_-mediated changes in vascular and endothelial dysfunction related pathways, leading to changes in renal function (7, 34). Different components of PM_2.5_ may have different toxic effects. It is hard to examine the difference between our results and previous ones because there are few studies on the effects of PM_2.5_ components on renal function indicators during pregnancy. In this study, the components with the first and second contribution weight to SCr at 13 weeks of gestation were OC and SOIL at 4 weeks before pregnancy, and SOIL and BC at 13 weeks of gestation. The difference in the contributions of the pollutant mixture components between the 4 weeks before pregnancy and at 13 weeks of gestation can be caused by various factors such as seasonal variation in pollution levels, changes in individual behavior or activities, developmental changes in the body's susceptibility to pollutants during pregnancy, and differences in air pollutant sources and composition over time (35, 36).

In China, about 50%−60% of urban PM_2.5_ comes from diesel vehicle exhaust emissions in transportation, civil cooking and heating, coal-fired industrial production, and biomass combustion. Carbon-containing components are the main PM_2.5_ components produced in the above industrial processes, including BC and OC. BC usually exists in the form of aggregates with a diameter of 85–500 nm, with small particle sizes (ranging from a few nanometers to several hundred nanometers). OC contains thousands of organic compounds, and these two types of carbon-containing components are closely related to human health (37–39). OC, particularly secondary organic aerosols and combustion-derived organics, has been reported to exhibit high oxidative potential and to induce systemic inflammation and endothelial dysfunction, which may impair renal microvascular function and glomerular filtration. These pathways may partly explain the stronger contribution of OC observed in our WQS models (40). SOIL aerosols contain a variety of heavy metals and endocrine disrupting chemicals (EDCs), such as polychlorinated biphenyls (PCBs), and polycyclic aromatic hydrocarbons. Evidence shows that SOIL can cause chronic or acute damage to mother and child (41). Soil-related particles may also act as carriers of transition metals and toxic organic compounds, which can generate reactive oxygen species and activate inflammatory signaling cascades after inhalation. Systemic inflammation and oxidative stress may subsequently affect renal tubular cells and glomerular structures, thereby contributing to alterations in renal biomarkers (42).

In mixture exposure studies, it is normal for different statistical models to yield slightly different results due to variations in their algorithmic assumptions. The WQS model is suitable for scenarios where effects are directionally consistent, whereas the Qgcomp model can simultaneously capture both positive and negative effects, making it more sensitive to mixtures with potential antagonistic interactions (43). In this study, the two models showed strong consistency in the core findings, and the discrepancies in secondary indicators precisely reflect the value of Qgcomp as a sensitivity analysis, as it not only validates the robustness of the main conclusions but also reveals potential complex interactions among PM_2.5_ components, offering clues for subsequent mechanistic research.

Some advantages can be observed in our study. Firstly, the study was conducted based on a prospective birth cohort with strict quality control, ensuring the results possess causal reliability. Secondly, we focused on the components of PM_2.5_, utilizing a satellite spatiotemporal inversion model to comprehensively evaluate the exposure to six major components of PM_2.5_. Thirdly, we first explored the effects of exposure to PM_2.5_ and its components before pregnancy, a period that has received less attention, on renal function indicators during pregnancy. Finally, we employed a novel approach by comparing multiple statistical models for environmental mixed exposure assessment to thoroughly evaluate the health impact of PM_2.5_ and component exposure on renal function during pregnancy.

This study has several limitations that should be noted. Firstly, the exposure levels of PM_2.5_ and its components were calculated based solely on participants' home addresses, without incorporating workplace exposure, which may have resulted in exposure misclassification. Secondly, some potential confounders were not available in this study, including temperature and relative humidity, as well as lifestyle-related factors such as outdoor exercise and dietary habits; these unmeasured or residual confounders may have influenced the observed associations. Thirdly, for the exposure window at 13 weeks of gestation, exposure assessment and renal function measurement were conducted at the same time point; therefore, these associations should be interpreted cautiously as cross-sectional and temporality cannot be fully established. Finally, exposure was assessed only during selected preconception windows (4, 8, and 12 weeks before pregnancy) and at 13 weeks of gestation; thus, longer-term cumulative exposure may not be fully captured, which should be considered when interpreting our findings.

## 5 Conclusions

In summary, the multiple linear regression and WQS regression models were employed to evaluate the correlation between air contamination and renal function indicators in pregnant women. Mixed exposure to PM_2.5_ components before and during pregnancy was related to changes in renal function in Chinese pregnant women, among which the most important components were OC, BC, and SOIL. The importance of combining different approaches to assess the effects of environmental chemicals on the kidney health of pregnant women must be emphasized.

## Data Availability

The datasets presented in this article are not readily available because The data cannot be shared publicly, in order to protect the privacy of study participants Requests to access the datasets should be directed to sepnine@njmu.edu.cn.
