# Supplementary material for: The impact of mixed exposure to PM2.5 components before and during pregnancy on renal function in pregnant women
Source: Front Public Health. 2026 May 20;14:1770917. doi: 10.3389/fpubh.2026.1770917 (PMC13230117; doi:10.3389/fpubh.2026.1770917)
Supplement: Supplementary file 1 [file Data_Sheet_1.docx]

**The Impact of Mixed Exposure to PM_2.5_ Components Before and During Pregnancy on Renal Function in Pregnant Women**

Wu Yan Ph.D ^1#^_,_ Xuemei Guo M.D ^2#^_,_ Yiming Du M.D ^3#^, Beibei Gao Ph.D ^4,5^_,_ Rufeng Lin Ph.D ^2*^ and Xu Wang Ph.D ^6*^

^1^ Department of Children Health Care, Children's Hospital of Nanjing Medical University, Nanjing, 210008, China. [yanwu@njmu.edu.cn](file:///D:\燕武99999999999999999999999999\大气污染与肾功能\投稿\14.%20Frontiers%20in%20public%20health\yanwu@njmu.edu.cn) (Wu Yan)

^2^ Department of Hematology and Oncology, Children's Hospital of Nanjing Medical University, Nanjing, 210008, China. [guoxuemei5531@163.com](mailto:guoxuemei5531@163.com) (Xuemei Guo); [linrufeng0907@sina.com](mailto:linrufeng0907@sina.com) (Rufeng Lin)

^3^ Pediatric Department, Zhongda Hospital, Southeast University, Nanjing, 210009, China. dymmzd@126.com (Yiming Du)

^4^ State Key Laboratory of Reproductive Medicine and Offspring Health, Center for Global Health, School of Public Health, Nanjing Medical University, Nanjing 211166, China, [gaob@njmu.edu.cn](file:///D:\燕武99999999999999999999999999\大气污染与肾功能\投稿\14.%20Frontiers%20in%20public%20health\gaob@njmu.edu.cn) (Beibei Gao)

^5^ Key Laboratory of Modern Toxicology of Ministry of Education, School of Public Health, Nanjing Medical University, Nanjing 211166, China

^6^ Clinical Medical Research Center, Children's Hospital of Nanjing Medical University, Nanjing, 210008, China. [sepnine@njmu.edu.cn](file:///D:\燕武99999999999999999999999999\大气污染与肾功能\投稿\14.%20Frontiers%20in%20public%20health\sepnine@njmu.edu.cn) (Xu Wang)

^#^ These authors have contributed equally to this work and share first authorship (Wu Yan, Xuemei Guo, and Yiming Du)

**Corresponding author**

Dr. Rufeng Lin. E-mail: [linrufeng0907@sina.com](mailto:linrufeng0907@sina.com)

Dr. Xu Wang. E-mail: [sepnine@njmu.edu.cn](mailto:sepnine@njmu.edu.cn)

**Table S1** The characteristics of pregnant women, and their exposure to PM_2.5_ during pregnancy

| Characteristic |  | PM2.5 (μg/m^3^) | *F* | *P*-values |
| --- | --- | --- | --- | --- |
|  | N | (MEAN±SD) |  |  |
| Maternal age (years) |  |  |  |  |
| <30 | 962 | 61.28±13.69 | 3.580 | **0.028** |
| 30-35 | 472 | 62.50±13.51 |  |  |
| >35 | 74 | 58.17±14.10 |  |  |
| Pre-pregnancy BMI (kg/m^2^) |  |  |  |  |
| <18.5 | 260 | 60.89±13.96 | 1.526 | 0.218 |
| 18.5-24.9 | 1092 | 61.41±13.56 |  |  |
| ≥25 | 156 | 63.23±14.00 |  |  |
| Maternal education |  |  |  |  |
| <high school | 176 | 59.17±14.05 | 3.009 | **0.050** |
| high school | 369 | 61.55±13.88 |  |  |
| >high school | 963 | 61.92±13.5 |  |  |
| Alcohol consumption |  |  |  |  |
| No | 1455 | 61.43±13.68 | -1.214 | 0.225 |
| Yes | 53 | 63.75±13.54 |  |  |
| Household income (yuan) |  |  |  |  |
| <30 thousand | 22 | 60.19±15.84 | 1.693 | 0.149 |
| 30-50 thousand | 106 | 59.78±13.44 |  |  |
| 50-100 thousand | 355 | 60.46±13.44 |  |  |
| 100-200 thousand | 823 | 61.87±13.61 |  |  |
| > 200 thousand | 202 | 62.94±14.15 |  |  |
| Twin |  |  |  |  |
| No | 1481 | 61.58±13.71 | 1.532 | 0.126 |
| Yes | 27 | 57.51±10.96 |  |  |
| Parity (times) |  |  |  |  |
| 0 | 1213 | 61.88±13.62 | 2.305 | 0.100 |
| 1 | 288 | 60.04±13.88 |  |  |
| 2 | 7 | 58.18±12.02 |  |  |
| Assisted reproduction |  |  |  |  |
| No | 1478 | 61.53±13.66 | 0.520 | 0.603 |
| Yes | 30 | 60.22±14.81 |  |  |

**Table S2.** Baseline characteristics of pregnant women included in and excluded from the uric acid (UA) analysis

| Characteristic | Excluded UA (N=809) | Included UA (N=699) | F | P-value |
| --- | --- | --- | --- | --- |
| Maternal age (years) |  |  |  |  |
| <30 | 481 | 481 | 19.941 | 0.001 |
| 30–35 | 274 | 198 |  |  |
| >35 | 54 | 20 |  |  |
| Pre-pregnancy BMI (kg/m²) |  |  |  |  |
| <18.5 | 146 | 114 | 8.454 | 0.015 |
| 18.5–24.9 | 564 | 528 |  |  |
| ≥25 | 99 | 57 |  |  |
| Maternal education |  |  |  |  |
| <High school | 71 | 105 | 24.542 | 0.001 |
| High school | 177 | 192 |  |  |
| >High school | 561 | 402 |  |  |
| Alcohol consumption |  |  |  |  |
| No | 780 | 675 | 0.025 | 0.874 |
| Yes | 29 | 24 |  |  |
| Household income (yuan) |  |  |  |  |
| <30 thousand | 10 | 12 | 34.702 | 0.001 |
| 30–50 thousand | 55 | 51 |  |  |
| 50–100 thousand | 172 | 183 |  |  |
| 100–200 thousand | 493 | 330 |  |  |
| >200 thousand | 79 | 123 |  |  |
| Passive smoking |  |  |  |  |
| No | 617 | 516 | 1.341 | 0.720 |
| <0.5 hours/day | 153 | 143 |  |  |
| 0.5–1 hours/day | 31 | 32 |  |  |
| >1 hour/day | 8 | 8 |  |  |
| Twin |  |  |  |  |
| No | 795 | 686 | 0.036 | 0.850 |
| Yes | 14 | 13 |  |  |
| Parity (times) |  |  |  |  |
| 0 | 667 | 546 | 11.161 | 0.004 |
| 1 | 142 | 146 |  |  |
| 2 | 0 | 7 |  |  |
| Assisted reproduction |  |  |  |  |
| No | 791 | 687 | 0.497 | 0.481 |
| Yes | 18 | 12 |  |  |

**Table S3** Distribution of air pollutants in the location of pregnant women.

|  | 25th | 50th | 75th | Range |
| --- | --- | --- | --- | --- |
| At 13 weeks of gestation (First trimester) | | | | |
| PM_2.5_ | 48.50 | 61.00 | 75.25 | (34.00-89.25) |
| OC | 5.78 | 8.70 | 18.70 | (3.70-29.93) |
| BC | 3.32 | 4.27 | 5.45 | (2.02-7.27) |
| SO_4_^2-^ | 12.75 | 14.62 | 16.08 | (8.05-28.23) |
| NH_4_^+^ | 9.03 | 10.30 | 11.38 | (6.90-15.33) |
| NO_3_^-^ | 11.95 | 15.90 | 20.99 | (7.33-29.20) |
| SOIL | 2.33 | 2.95 | 3.58 | (0.27-13.47) |
| 4 weeks before pregnancy | | | | |
| PM_2.5_ | 56.10 | 59.00 | 63.42 | (46.89-75.50) |
| OC | 10.73 | 12.09 | 13.34 | (5.94-15.29) |
| BC | 3.98 | 4.27 | 4.70 | (3.07-5.49) |
| SO_4_^2-^ | 12.52 | 14.76 | 15.48 | (10.76-19.03) |
| NH_4_^+^ | 9.43 | 9.69 | 10.33 | (8.28-12.29) |
| NO_3_^-^ | 15.00 | 16.65 | 17.66 | (11.42-19.76) |
| SOIL | 2.69 | 3.02 | 3.43 | (1.89-5.66) |
| 8 weeks before pregnancy | | | | |
| PM_2.5_ | 43.00 | 54.50 | 69.00 | (25.00-111.00) |
| OC | 4.50 | 7.30 | 17.45 | (2.50-42.20) |
| BC | 2.60 | 4.00 | 5.50 | (1.70-8.60) |
| SO_4_^2-^ | 11.60 | 13.70 | 15.70 | (5.60-28.40) |
| NH_4_^+^ | 8.00 | 9.20 | 10.70 | (4.90-16.10) |
| NO_3_^-^ | 8.80 | 14.20 | 22.60 | (4.7-39.70) |
| SOIL | 0.60 | 2.40 | 4.90 | (0.10-13.60) |
| 12 weeks before pregnancy | | | | |
| PM_2.5_ | 43.25 | 54.50 | 71.50 | (28.00-102.00) |
| OC | 4.55 | 7.42 | 16.95 | (3.20-36.45) |
| BC | 2.65 | 4.00 | 5.25 | (1.80-7.55) |
| SO_4_^2-^ | 12.15 | 13.50 | 15.45 | (6.10-23.85) |
| NH_4_^+^ | 8.05 | 9.45 | 10.90 | (5.40-13.80) |
| NO_3_^-^ | 8.75 | 14.25 | 22.53 | (5.80-34.30) |
| SOIL | 0.80 | 3.15 | 5.50 | (0.30-14.50) |

PM_2.5_: particulate matter 2.5 (μg/m^3^), OC: organic matter (μg/m^3^), BC: black carbon (μg/m^3^),

SO_4_^2-^: sulfate (μg/m^3^), NH_4_^+^: ammonium salt (μg/m^3^), NO_3_^-^: nitrate (μg/m^3^), SOIL: soil dust (μg/m^3^)

**Table S4** Distribution of renal function in pregnant women.

|  | 25th | 50th | 75th | Range |
| --- | --- | --- | --- | --- |
| BUN (n=1499) | 2.39 | 2.82 | 3.36 | (1.00-9.00) |
| SCr (n=1508) | 37.50 | 43.90 | 50.10 | (3.55-208.50) |
| UA (n=699) | 171.4 | 195.4 | 224.7 | (57.70-478.30) |

BUN: Blood urea nitrogen (mmol/L); SCr: Serum Creatinine (μmol/L); UA: Uric acid (μmol/L)

**Table S5.** Associations between the mixture of PM_2.5_ components and renal function among pregnant women using Qgcomp

|  | Ψ (95%CI) | *P*-value | Direction | Weight | | | | | |
| --- | --- | --- | --- | --- | --- | --- | --- | --- | --- |
|  |  |  |  | OC | BC | SO_4_^2-^ | NH_4_^+^ | NO_3_^-^ | SOIL |
| At 13 weeks of gestation (First trimester) | | | | | | | | | |
| BUN | 0.07 (-0.022, 0.161) | 0.135 | pos |  |  | 0.145 |  | 0.792 | 0.062 |
|  |  |  | neg | 0.500 | 0.389 |  | 0.111 |  |  |
| SCr | 0.383 (-0.926, 1.693) | 0.566 | pos |  | 0.165 |  | 0.132 | 0.364 | 0.338 |
|  |  |  | neg | 0.632 |  | 0.368 |  |  |  |
| UA | -0.292 (-7.254, 6.67) | 0.935 | pos |  |  | 0.292 | 0.050 | 0.658 |  |
|  |  |  | neg | 0.325 | 0.366 |  |  |  | 0.310 |
|  |  |  |  |  |  |  |  |  |  |
| 4 weeks before pregnancy | | | | | | | | | |
| BUN | -0.047 (-0.127, 0.033) | 0.254 | pos |  | 0.755 |  |  | 0.015 | 0.230 |
|  |  |  | neg | 0.585 |  | 0.231 | 0.184 |  |  |
| SCr | 3.392 (2.281, 4.503) | ＜0.001 | pos | 0.439 | 0.110 | 0.004 |  | 0.219 | 0.228 |
|  |  |  | neg |  |  |  | 1.000 |  |  |
| UA | 7.575 (2.208, 12.942) | 0.006 | pos |  |  | 0.319 |  | 0.652 | 0.029 |
|  |  |  | neg | 0.261 | 0.191 |  | 0.548 |  |  |
| 8 weeks before pregnancy | | | | | | | | | |
| BUN | -0.051 (-0.117, 0.016) | 0.137 | pos |  | 0.594 |  | 0.251 | 0.155 |  |
|  |  |  | neg | 0.159 |  | 0.520 |  |  | 0.322 |
| SCr | -1.524 (-2.832, -0.216) | 0.023 | pos |  | 0.175 |  | 0.412 | 0.119 | 0.294 |
|  |  |  | neg | 0.419 |  | 0.581 |  |  |  |
| UA | 7.089 (0.886, 13.291) | 0.025 | pos | 0.032 |  | 0.033 | 0.307 | 0.456 | 0.173 |
|  |  |  | neg |  | 1.000 |  |  |  |  |
| 12 weeks before pregnancy | | | | | | | | | |
| BUN | -0.076 (-0.151, -0.001) | 0.049 | pos |  | 1.000 |  |  |  |  |
|  |  |  | neg | 0.234 |  | 0.358 | 0.059 | 0.126 | 0.223 |
| SCr | -2.654 (-4.043, -1.265) | ＜0.001 | pos |  | 0.247 |  | 0.434 |  | 0.319 |
|  |  |  | neg | 0.310 |  | 0.541 |  | 0.149 |  |
| UA | 7.403 (-0.12, 14.927) | 0.054 | pos | 0.481 | 0.110 | 0.023 | 0.210 |  | 0.175 |
|  |  |  | neg |  |  |  |  | 1.000 |  |

BUN: Blood urea nitrogen (mmol/L); SCr: serum creatinine (umol/L); UA: Uric acid (μmol/L)

CI: confidence interval.

The model adjusted for age, BMI, education, passive smoking, alcohol consumption, household income, twin, parity, and assisted reproduction.

In BUN, SCr, and UA model analyses, there were 1499, 1508, and 699 participants, respectively.

**
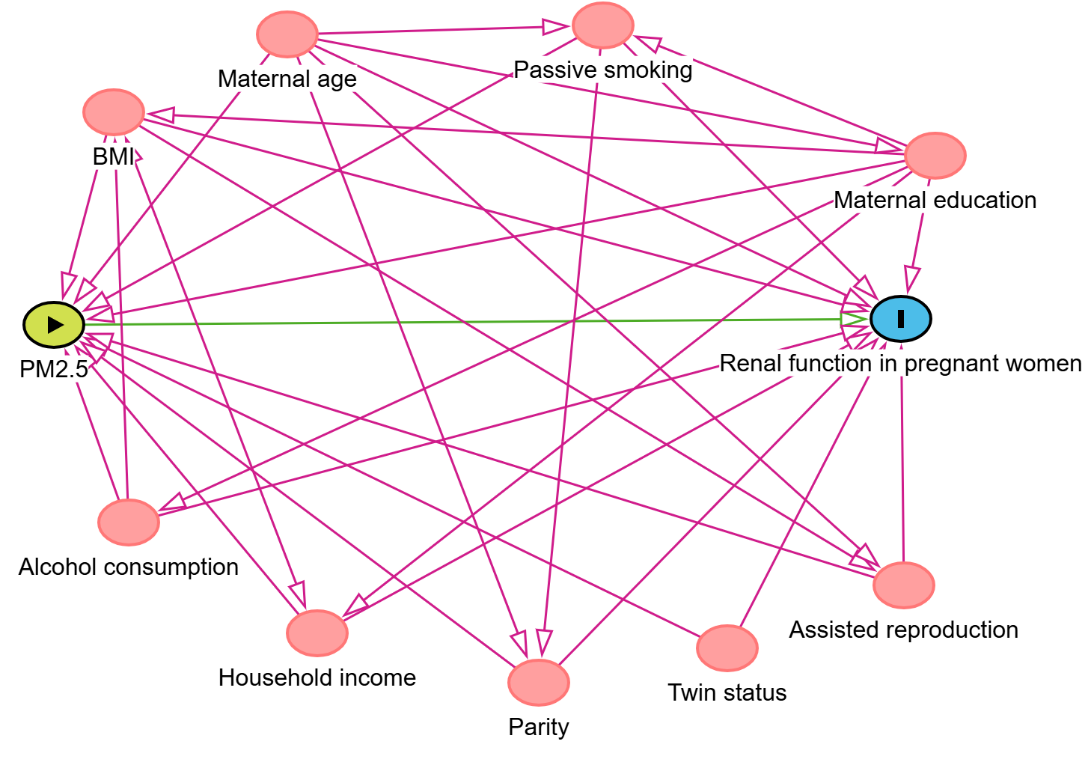
**

**Figure S1.** Directed acyclic graph (DAG) for visualizing the minimum adjustement set of caovariates selected
